# White matter integrity in narcolepsy: the structural blueprint for functional complaints?

**DOI:** 10.1093/sleep/zsae020

**Published:** 2024-01-23

**Authors:** Jari K Gool, Thien Thanh Dang-Vu, Ysbrand D van der Werf

**Affiliations:** Anatomy and Neurosciences, Amsterdam UMC location Vrije Universiteit Amsterdam, Amsterdam, The Netherlands; Stichting Epilepsie Instellingen Nederland (SEIN), Sleep-Wake Centre, Heemstede, Netherlands; Department of Neurology, Leiden University Medical Centre, Leiden, The Netherlands; Compulsivity, Impulsivity and Attention, Amsterdam Neuroscience, Amsterdam, The Netherlands; Centre de recherche de l’Institut universitaire de gériatrie de Montréal, Centre intégré universitaire de santé et de services sociaux du Centre-Sud-de-l’Ile-de-Montréal, Montreal, QC, Canada; Center for Studies in Behavioral Neurobiology, Department of Health, Kinesiology and Applied Physiology, Concordia University, Montreal, QC, Canada; Anatomy and Neurosciences, Amsterdam UMC location Vrije Universiteit Amsterdam, Amsterdam, The Netherlands; Compulsivity, Impulsivity and Attention, Amsterdam Neuroscience, Amsterdam, The Netherlands

**Figure 1.**
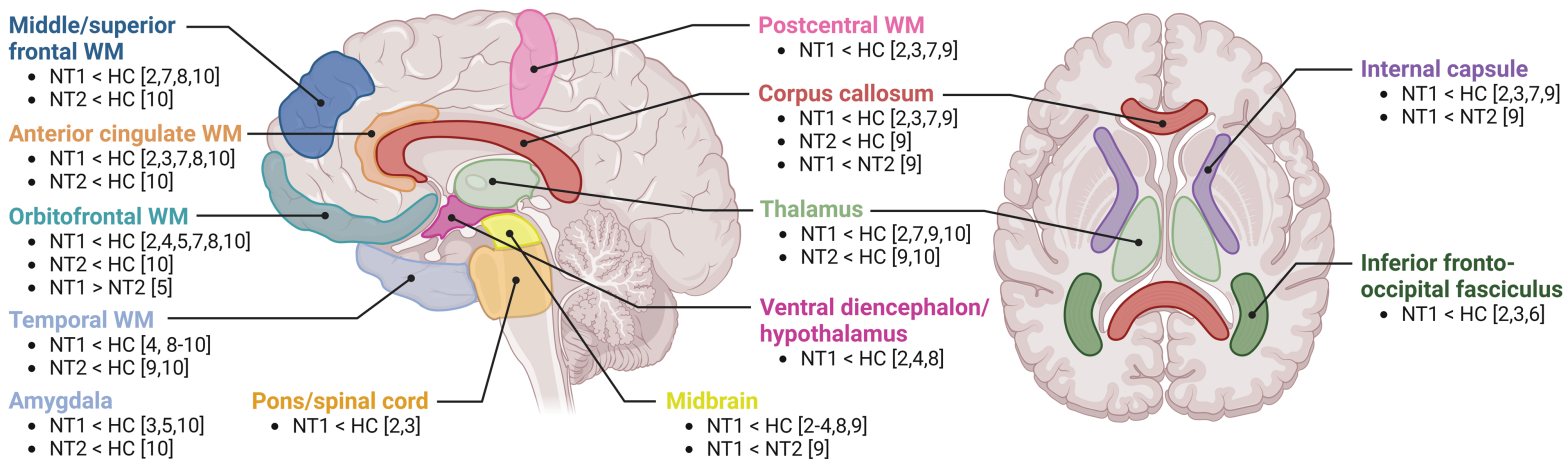
Summary figure of diffusion-weighted imaging (DWI) studies investigating structural connectivity in narcolepsy. Regions were generally included in case there were at least three independent studies indicating white matter changes within this region in people with narcolepsy compared to controls. Reported results were limited to reduced structural connectivity in narcolepsy as the vast majority of studies solely reported reduced structural connectivity. Reported differences could reflect any DWI outcome measure that represents white matter integrity, such as fractional anisotropy (FA) or mean diffusivity/apparent diffusion coefficient (MD/ADC). The numbers between brackets correspond to the reference numbers of the studies. The study by Chen et al. [10] did not divide narcolepsy into type 1 and type 2 when comparing structural connectivity to controls. Post hoc comparisons between narcolepsy type 1 and type 2 did not reveal differences between narcolepsy subtypes, and the study by Chen et al. [10] was thus reported for both the “NT1 < HC” and the “NT2 < HC” contrasts. HC, healthy controls; NT1, narcolepsy type 1; NT2, narcolepsy type 2; WM, white matter.

Narcolepsy is a chronic neurological sleep disorder characterized by excessive daytime sleepiness and is generally divided into two subtypes: narcolepsy type 1 and type 2. Besides the daytime sleepiness with sleep attacks, both narcolepsy subtypes show sleep-onset rapid eye movement (REM) sleep periods and are frequently accompanied by hypnagogic/hypnopompic hallucinations, sleep paralysis, disturbed nocturnal sleep, vigilance deficits, and in case of narcolepsy type 1, cataplexy. Narcolepsy type 1 is caused by hypocretin-1 deficiency, whereas the etiology of narcolepsy type 2 remains unknown [1]. The multitude of affected symptom domains in narcolepsy has interested many research groups to investigate underlying neural signatures. Using diffusion-weighted imaging (DWI), studies have suggested widespread structural white matter differences in narcolepsy type 1 compared to controls in practically all brain regions (Figure 1) [2–9].

Chen and colleagues are the first to multimodally combine white matter assessment in narcolepsy with healthy individuals’ genetic expression and glutamatergic PET/SPECT profiles [10]. Limited drug-naïve samples of both narcolepsy subtypes were included in the DWI analyses, which showed generalized reduced structural connectivity, which also correlated with shorter objective sleep latency. Comparisons between narcolepsy type 1 and type 2 did not show significant differences in connectivity between subtypes, which contrasts with the few previous structural magnetic resonance imaging (MRI) studies comparing these populations (Figure 1) [5, 9, 11, 12]. This could be due to the small sample sizes included by Chen and colleagues, or indicate that the observed alterations are consequential to excessive daytime sleepiness instead of being specific for hypocretin deficiency. In the absence of hypocretin-1 measurements, it is also possible that hypocretin-1 deficiency without cataplexy could have led to misdiagnosis of narcolepsy type 2 in some individuals. Cautious interpretation of the genetic transcription and glutamatergic positron emission tomography-single photon emission computed tomography (PET-SPECT) findings is needed as these analyses relied exclusively on an external healthy population, lacking individuals with narcolepsy. The suggested pathophysiological relationship between glutamatergic signaling and narcolepsy mainly underscores the dire need for magnetic resonance spectroscopy studies, as neurometabolite concentrations in this context have been minimally explored [13, 14]. Future studies should combine larger groups of both narcolepsy types with idiopathic hypersomnia, and include control groups of sleep-deprived individuals to assess the specificity of the structural connectivity findings.

Symptoms of REM sleep behavior disorder (RBD) are common in narcolepsy. Chen and colleagues report high RBD prevalences of 71% in narcolepsy type 1 (30%–60% in previous studies) and 54% in narcolepsy type 2 (15%–40% in previous studies) [15]. In narcolepsy with RBD, a structurally enhanced subnetwork of the thalamus, striatum, and cingulate gyrus was identified [10]. These regions are usually activated during REM sleep but are not known for mediating REM sleep muscle atonia. In idiopathic RBD, REM sleep without atonia arises from the dysfunctioning of REM-regulating pontomedullary nuclei, primarily through glutamatergic neurons in the subcoeruleus [16]. Changes in neuron counts outside the hypothalamus have so far not been identified in narcolepsy type 1 [17, 18], and loss of hypocretinergic innervation of brainstem REM circuits and the basal ganglia seems a more reasonable hypothesis for RBD symptoms in narcolepsy type 1. Different underlying pathophysiology could account for the nuanced distinctions in RBD phenotypes, such as the typically younger onset in narcolepsy compared to idiopathic RBD [15]. This could also clarify why, until now, narcolepsy has not shown an association with elevated incidence rates of alpha-synucleinopathies.

The exact relationship between the structural and functional differences observed in narcolepsy type 1 remains a chicken or egg debate. Multiple resting-state and task-based functional MRI (fMRI) studies have identified extensive functional connectivity differences in narcolepsy type 1, including the default mode (DMN), limbic and attention networks [19–22]. Most consistent findings comprise altered limbic activity during emotional processing [23–25], and the compromised capacity of the DMN, the primary resting network, to disengage from active networks when cognitively challenged. This inability of the DMN to decouple from other networks suggests a dysregulation in narcolepsy type 1 in favor of staying awake over actual task performance [26]. Understanding whether structural distinctions directly contribute to altered brain activity or if they are an outcome of prolonged symptomatology remains ambiguous, as most studies focused on individuals with long disease durations. A plausible perspective is that the observed structural effects are intricately tied to the chronic symptoms associated with hypocretin deficiency. Further investigations should focus on individuals in early disease stages to gain a more comprehensive understanding.

Can we conclude that people with narcolepsy have less efficient structural brain wiring? DWI uses indirect measures of water diffusion to assess white matter integrity and presumes that diffusion is relatively unrestricted along axon bundles compared to directions perpendicular to the axonal bundles. From conventional DWI findings, it remains unclear whether myelin and/or axonal integrity are affected [27, 28], or whether other processes involving water diffusion play an important mediating role. Recent studies revealed that the brain undergoes clearance of waste metabolites through cerebrospinal fluid (CSF) flow during sleep [29]. The effects of disturbed sleep on brain clearance (and possibly water diffusion) remain unknown and require further investigations, especially in narcolepsy, where there are ample diurnal and nocturnal sleep–wake alterations [1]. Future research is essential before arriving at conclusive findings. Specifically, the incorporation of postmortem immunohistochemistry analyses or multishell DWI studies could offer resolutions for accurately interpreting the widespread DWI alterations in narcolepsy.

Compared to many other neurological and psychiatric disorders, neuroimaging in narcolepsy and idiopathic hypersomnia remains in its early stages with limited multimodal perspectives, few cross-disorder comparisons, and typically small sample sizes leading to inconsistent findings. Both technically advanced studies generating new hypotheses and large-scale international collaborations verifying these results are necessary to overcome these limitations. The multimodal methodology of Chen and colleagues guides future more extensive studies in which clearly distinct narcolepsy subtypes and controls will be included for all acquisition methods [10]. To confront the challenge of inconsistent outcomes, our recently established neuroimaging consortium on central disorders of hypersomnolence (NICHY) presents a possible solution [30]. This initiative involves the global integration of existing and future MRI datasets related to narcolepsy and idiopathic hypersomnia to conduct cross-disorder comparisons among hypersomnolence diagnoses with unprecedented statistical power. Where first analyses focus on T1-weighted structural measures, future projects will also target DWI-derived structural and fMRI connectivity measures.

In conclusion, the question of whether disturbed white matter integrity in narcolepsy serves as a blueprint for functional complaints sparks an interesting debate. The findings of Chen and colleagues suggest a potential link between structural alterations and narcolepsy symptomatology that requires further investigation. Despite important methodological limitations to consider when interpreting findings by Chen and colleagues, the study illustrates the bright future of neuroimaging research in narcolepsy, where first efforts are being made to link brain structure with multimodal functioning [10]. Important questions remain: Are changes in DWI metrics in narcolepsy attributed to myelin, axonal density, or other poorly understood diffusion processes? To what degree are the extensive variations specific to hypocretin deficiency, or do these findings result from chronic exposure to excessive daytime sleepiness? What is the relationship between brain structure and functioning in narcolepsy? More multimodal efforts and upcoming multi-center collaborations like NICHY will be essential to build a comprehensive understanding of the neural signatures of central disorders of hypersomnolence.
